# From ABCD to AI: Assessing the Diagnostic Reliability of MLLMs in Cutaneous Melanoma Screening—A Head-to-Head Comparison

**DOI:** 10.3390/diagnostics16071077

**Published:** 2026-04-02

**Authors:** Răzvan Ioan Andrei, Aniela Roxana Nodiți-Cuc, Silviu Cristian Voinea, Cristian Ioan Bordea, Alexandru Blidaru

**Affiliations:** 1 Department of General Surgery, “Carol Davila” University of Medicine and Pharmacy, B-dul Eroii Sanitari 8, 050474 Bucharest, Romania; ioan-razvan.andrei@umfcd.ro (R.I.A.); silviu.voinea@umfcd.ro (S.C.V.); cristian.bordea@umfcd.ro (C.I.B.); alexandru.blidaru@umfcd.ro (A.B.); 2 Department of Surgical Oncology, Institute of Oncology “Prof. Dr. Al. Trestioreanu”, Şos. Fundeni 252, 022328 Bucharest, Romania

**Keywords:** melanoma, Artificial Intelligence, MLLM, GPT-5, Gemini 3, Grok 4, diagnostic accuracy, dermatological screening

## Abstract

**Background**: Melanoma remains a leading cause of cancer-related mortality, with early detection being the primary determinant of survival. The emergence of MLLMs offers a potential paradigm shift in accessible screening. However, the diagnostic reliability and safety of these general-purpose models in oncology remain insufficiently characterized. **Methods**: This study performed a head-to-head comparison of GPT-5, Gemini 3, and Grok 4 to evaluate their efficacy as first-level screening tools for cutaneous melanoma. A retrospective analysis was conducted using a balanced dataset of 100 clinical images (50 histopathologically confirmed benign, 50 malignant) from the ISIC archive. **Results**: Gemini 3 achieved the highest overall accuracy (71%) and specificity (94%), while Grok 4 demonstrated the highest sensitivity (52%). All models exhibited a critical deficit in sensitivity, missing approximately half of the malignant lesions. Statistical testing revealed no significant performance differences between the models (*p* > 0.05). Notably, Gemini 3 exhibited severe overconfidence, maintaining a high CI (84.62%) even during false-negative predictions, whereas GPT-5 and Grok 4 showed better calibration with a significant drop in confidence upon incorrect diagnosis. **Conclusions**: While current MLLMs possess a foundational capacity for dermatological analysis, their unacceptably low sensitivity and potential for overconfident misdiagnosis render them unsafe as standalone screening tools. At present, MLLMs should only be utilized as complementary tools under strict clinical supervision.

## 1. Introduction

Currently ranking as the fifth most frequent malignancy in the United States and sixth in Europe, melanoma is characterized by an epidemiological surge that outpaces all other forms of cancer [1,2]. This escalating incidence underscores a critical clinical reality: the timing of diagnosis is the primary determinant of both patient survival and the subsequent economic burden on the healthcare system. While the localized melanoma five-year survival rate is approaching 100%, this goes down to just 35% once the disease progresses to distant metastasis [3]. Furthermore, the healthcare fiscal implications of delayed detection are staggering, as advanced-stage malignancies account for more than 90% of all melanoma-related expenditures [4]. In this moment it is estimated that only half of the newly discovered cases are initially identified by specialized dermatologists [5]. This diagnostic gap highlights an urgent need for accessible screening tools that can support non-specialists and patients in identifying suspicious lesions at their most treatable stage.

For nearly four decades, the ABCD rule and the “ugly duckling” sign have served as the foundational pillars of skin cancer screening and were designed to give clinicians and patients a standardized way to evaluate pigmented lesions. The ABCD rule was first proposed in 1985 by a team at New York University led by Dr. Alfred Kopf, which included Dr. Darrell Rigel and Dr. Robert Friedman. They argued that the combination of routine physician examination of the skin coupled with self-examination provides a realistic opportunity for early detection, which can significantly reduce the mortality rate [6]. Originally, it was just ABCD; the E (for evolving) was added later around 2004 as doctors realized that any mole that changes over time is one of the strongest indicators of malignancy [7]. While this rule looks at moles in isolation, the “ugly duckling” sign takes a bigger-picture approach. This concept was introduced in 1998 by Dr. Jean-Jacques Grob and his colleagues. The idea is based on the observation that a person’s moles tend to look alike—they usually share a similar pattern, color, and shape [8]. Instead of checking if a mole meets the classic ABCD criteria, you look at all the moles on a specific area of the body, and if one mole looks significantly different from all the others around it, then it is the “ugly duckling” and should be checked [8]. It accounts for people who have many atypical-looking moles naturally, because if all moles are large and fuzzy, a small, dark, neat mole might actually be the dangerous one [8].

These rules were designed for simplicity, refining the complex morphology of melanocytic lesions into a checklist for laypersons and General Practitioners (GPs) [7]. In this model, the observer looks for macro-architectural features like asymmetry or diameter > 6 mm, effectively filtering out any data that does not fit these human-defined categories [6]. What one clinician defines as a blurred border, another may see as regular, leading to significant inter-observer variability [9]. While excellent for detecting advanced lesions, these rules often fail to identify early-stage or featureless melanomas that have not yet developed the classic, chaotic hallmarks required to trigger a human alarm [10].

The paradigm of dermatological screening has been shifted by the emergence of Artificial Intelligence (AI). Initial deep learning approaches, primarily based on Convolutional Neural Networks (CNNs), demonstrated diagnostic accuracies comparable to, and occasionally exceeding, those of board-certified dermatologists in controlled environments [11]. However, these models often lack the ability to provide clinical reasoning. The emergence of Multimodal Large Language Models (MLLMs), such as GPT-4, represents a pivotal step in this field [12]. Unlike their unimodal predecessors—Large Language Models (LLMs),MLLMs possess the unique capacity to process both high-resolution clinical images and structured textual data simultaneously, generating comprehensive diagnostic narratives that mimic human clinical reasoning [13]. Despite their promise, the diagnostic reliability rates of these general-purpose models in the high-stakes domain of oncology remain insufficiently characterized.

This study aims to address this gap by providing a head-to-head comparison between state-of-the-art MLLMs. By evaluating their performance across a diverse spectrum of melanocytic lesions, we seek to determine whether these advanced AI models can serve as a reliable first-level screening. We aim that by measuring diagnostic capability, provide a quantitative calibration analysis to identify ‘blind overconfidence’ in oncological misdiagnosis and establish a performance baseline for general-purpose AI against histopathologically verified clinical data.

## 2. Materials and Methods

### 2.1. Input Data Selection

This study was designed as a retrospective comparative analysis of three state-of-the-art MLMMs in the context of cutaneous melanoma diagnosis. A dataset comprising 100 images of skin lesions was randomly selected from the ISIC 2024 Archive (International Skin Imaging Collaboration). To ensure the highest level of ground-truth reliability, we applied a strict inclusion filter: only lesions with histopathologically confirmed diagnoses were selected. The dataset was manually selected to ensure a 1:1 balance (50 malignant, 50 benign) and to verify that all images were ‘clinical close-ups’ with adequate focus and lighting, simulating a high-quality smartphone capture by a layperson or GP. Original ISIC image identifiers (IDs) were anonymized and randomized to prevent the AI models from relying on pre-existing diagnostic information associated with those specific identifiers.

### 2.2. MLLMs Selection and Methodology

Our study initially aimed to benchmark a wide array of MLLMs available in early 2026. However, during the experimental phase, we encountered significant safety alignment barriers. Several high-tier models—specifically GPT 5.4, 5.3, 5.1, Claude Sonnet 4.6, Claude Opus 4.5, Claude 4.1, and Gemini 3.1 Pro—consistently refused to provide binary diagnostic classifications or numeric probability scores. These models cited ethical constraints and safety guardrails regarding the provision of medical diagnoses from images, despite the inclusion of academic disclaimers and the use of anonymized public datasets. Therefore, we selected: GPT-5 (OpenAI, San Francisco, CA, USA), Gemini 3 (Google, Mountain View, CA, USA), and Grok 4 (xAI, Palo Alto, CA, USA) which could be made compliant with forced choice research labeling that in our case easily escaped the safety protocols for these models (Table 1). Each model was independently presented with the same set of 100 images. For each image, the models were prompted to output two specific variables: a classification of either benign or malignant and a Confidence Index (CI), a self-reported metric ranging from 0.0 to 1.0, representing the model’s certainty regarding its chosen binary diagnosis. It is important to note that the CI reflects diagnostic confidence, not the direct probability of malignancy. Models were accessed via a standardized multimodal interface (https://use.ai/, accessed on 14–16 March 2026), ensuring access to all models above-mentioned. To eliminate intra-session bias and context contamination, each of the 100 images was processed in a fresh, isolated session with immediate cache clearance post-inference. This protocol simulates a ‘zero-shot’ real-world diagnostic scenario while maintaining statistical independence between trials. To ensure methodological transparency and address the inherent complexity of benchmarking generative AI in a clinical context, our study adheres to the METRICS (Model, Evaluation, Timing, Range, Individual factors, Count, Specificity) framework. This standardized reporting structure was adopted to provide a reproducible trail of the diagnostic process [14]. By utilizing the METRICS checklist (Table 2), we explicitly define the computational environment, the parameters of the models and the exact constraints of the prompting strategy.

### 2.3. Statistical Analyses

Standard diagnostic performance metrics, including Overall Accuracy, Sensitivity and Specificity, were calculated for each model. To assess model calibration, the mean Confidence Index was compared between correct and incorrect predictions. To construct Receiver Operating Characteristic (ROC) curve and calculate the Area Under the Curve (AUC), the confidence scores were mathematically transformed into continuous probabilities of malignancy (a benign diagnosis with 80% confidence was converted to a 20% probability of malignancy). Differences in diagnostic accuracy between the paired models were evaluated for statistical significance using the exact McNemar’s test. A *p*-value of < 0.05 was considered statistically significant.

## 3. Results

### 3.1. Diagnostic Performance

**Figure 1:**
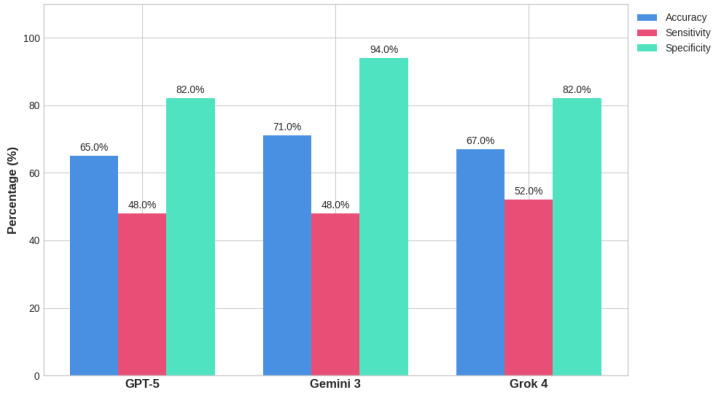
Performance Comparison: Accuracy, Sensitivity and Specificity.

A total of 100 skin lesion images (50 benign, 50 malignant) were evaluated. The diagnostic performance metrics for all three models are summarized in Figure 1. Gemini 3 achieved the highest overall accuracy at 71.0%, primarily driven by an outstanding specificity of 94.0%. However, all three models demonstrated significant limitations in detecting malignancy. Grok 4 exhibited the highest sensitivity at 52.0%, while both GPT-5 and Gemini 3 correctly identified only 48.0% of the malignant cases (24 out of 50).

Consequently, a high rate of false-negative predictions was common across all platforms. To gain a deeper understanding of the distribution of diagnostic errors, confusion matrices were generated (Table 3). The analysis revealed a disproportionate tendency across all models to underdiagnose cutaneous melanoma.

Gemini 3 demonstrated a highly conservative approach, producing only 3 FP results (meaning it rarely misclassified a benign lesion as a cancer), but at the severe cost of generating 26 FN results. Both Grok 4 and GPT-5 exhibited a slightly more aggressive diagnostic pattern, each producing 9 FP. However, they still failed to identify a significant portion of the malignant lesions, with Grok 4 generating 24 FN and GPT-5 matching Gemini 3 with 26 FN. In a clinical context, this high volume of false negatives represents a critical safety limitation, as melanomas would be left undiagnosed by the AI.

### 3.2. Statistical Comparison

Despite the apparent variations in overall accuracy (Gemini 3: 71%, Grok 4: 67%, GPT-5: 65%), the McNemar’s test revealed no statistically significant differences in the diagnostic performance between any of the model pairs: Gemini 3 vs. GPT-5 (*p* = 0.2101), Gemini 3 vs. Grok 4 (*p* = 0.5034), and GPT-5 vs. Grok 4 (*p* = 0.6875). This indicates a comparable baseline capability among the evaluated models for this specific visual task.

**Figure 2:**
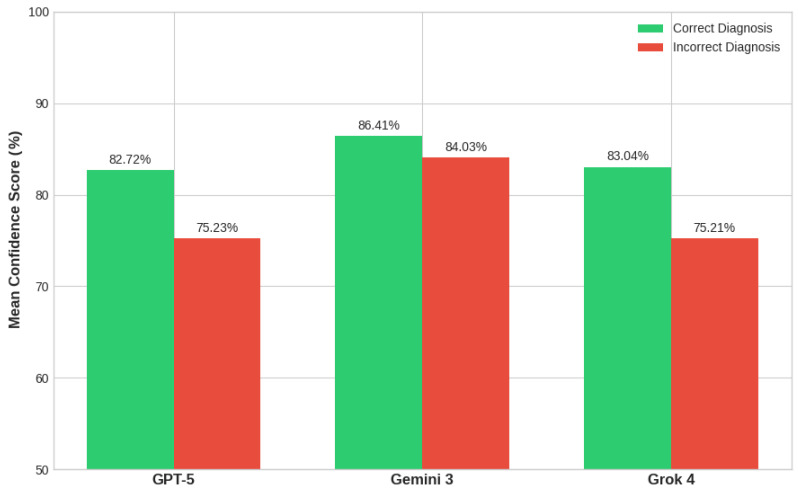
Calibration Analysis: Confidence on Correct vs. Incorrect Diagnoses.

However, analysis of the models self-reported CI revealed distinct calibration profiles (Figure 2). GPT-5 and Grok 4 demonstrated better calibration, showing a noticeable decrease in confidence when making incorrect diagnoses. Specifically, Grok 4 mean confidence dropped from 83.04% (correct diagnoses) to 75.21% (incorrect diagnoses), and GPT-5’s dropped from 82.72% to 75.23%. Conversely, Gemini 3 exhibited substantial overconfidence. There was a negligible difference in Gemini 3’s certainty between its correct predictions (CI: 86.41%) and its errors (CI: 84.03%). Alarmingly, when Gemini 3 missed a malignant lesion (false negative), it did so with a highly confident average score of 84.62%.

To overcome the limitations of simple accuracy and provide a more nuanced evaluation of diagnostic reliability, we expanded our statistical framework using three advanced metrics (Table 4). First, the F1-Score was calculated as the harmonic mean of precision and sensitivity, offering a balanced view of the trade-off between missing malignancies (FN) and over-diagnosing benign lesions (FP). Second, we calculated the Matthews Correlation Coefficient (MCC), which is widely regarded as the most robust balanced measure for binary classification, as it incorporates all four quadrants of the confusion matrix. Finally, to address the calibration gap, we utilized the Brier Score (BS). This metric quantifies the accuracy of the models’ self-reported probability estimates (Confidence Index); a lower Brier Score indicates that a model’s confidence levels are well-aligned with actual clinical outcomes, whereas a high score penalizes ‘overconfident’ misdiagnoses

The performance analysis reveals a significant discrepancy between raw accuracy and clinical reliability. While Gemini 3 achieved the highest F1-Score (0.623) and MCC (0.468), indicating superior general classification among the tested models, it simultaneously yielded the worst calibration profile with a Brier Score of 0.248. This statistical paradox is clinically critical: Gemini 3’s high penalty in the Brier Score stems from its tendency to assign high confidence levels (often >80%) to incorrect diagnoses, particularly in false-negative cases. In contrast, Grok 4 demonstrated the most reliable probabilistic mapping with the lowest Brier Score (0.217), suggesting that its internal confidence estimates are more representative of the actual diagnostic risk.

### 3.3. Concordance and Hard Cases

An analysis of inter-model agreement highlighted the intrinsic difficulty of the dataset. A striking total of 20 malignant lesions (40% of all malignant cases) were unanimously misclassified as benign by all three models (unanimous false negatives). In contrast, unanimous false positives were rare, with only a single benign lesion being incorrectly classified as malignant by all three AI systems.

**Figure 3:**
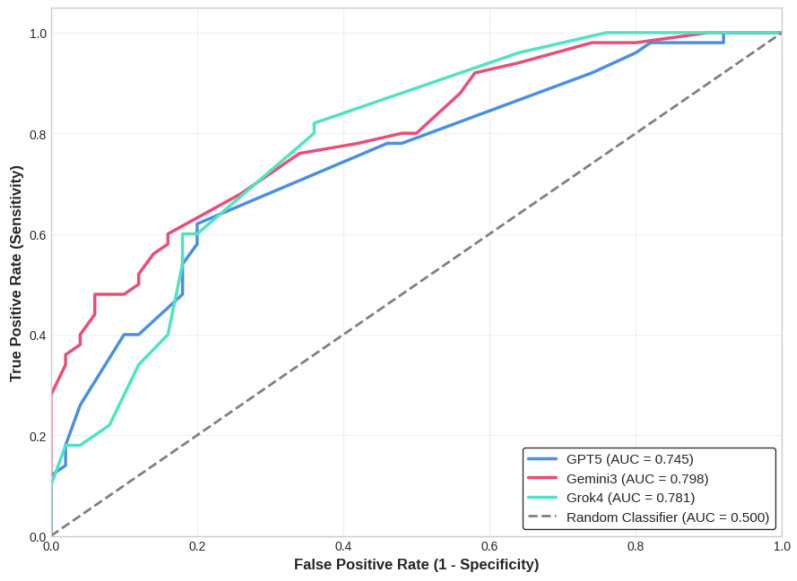
ROC Curves and AUC.

To further evaluate the discriminatory ability of the models beyond fixed binary thresholds, ROC curves were constructed, and the AUC was calculated (Figure 3). The self-reported CI were converted into continuous probabilities of malignancy to plot the curves. The ROC analysis demonstrated that all three models possess a moderate to good ability to distinguish between benign and malignant skin lesions. Gemini 3 achieved the highest discriminatory performance with an AUC of 0.798. It was closely followed by Grok 4, which yielded an AUC of 0.781. GPT-5 demonstrated the lowest overall discriminatory capacity among the tested models, with an AUC of 0.745. While Gemini 3’s high AUC reflects its superior specificity (as evidenced by its steep initial ROC curve), the clustering of the AUC values between 0.74 and 0.80 suggests that all models face similar underlying challenges when parsing complex dermatological visual data, failing to reach the highly accurate threshold (AUC > 0.90) typically required for valuable clinical diagnostic tools.

## 4. Discussion

The primary objective of this study was to evaluate whether contemporary general-purpose MLLMs can reliably serve as first-level screening tools for cutaneous melanoma. Despite demonstrating moderate overall discriminatory ability (AUCs ranging from 0.745 to 0.798) and high specificity (up to 94.0% for Gemini 3), our findings reveal a critical vulnerability: an unacceptably low sensitivity across all tested models. With the highest sensitivity reaching only 52.0% (Grok 4), these advanced AI systems missed approximately half of the malignant lesions.

In the clinical context of oncology, a screening tool must prioritize sensitivity to minimize FN, as a missed melanoma diagnosis directly correlates with a drop in survival rates [3]. The high specificity observed, particularly in Gemini 3, suggests that these models are heavily biased toward a benign baseline, requiring overwhelming visual evidence of malignancy to trigger a positive classification. While this minimizes unnecessary biopsies, it completely undermines the core purpose of early detection, rendering these models currently unsafe for independent patient use.

The clinical implications of our findings are profound. A false-negative diagnosis in melanoma is not merely a statistical error but a clinical catastrophe. Even a 3-to-6 month delay in diagnosis can precipitate a stage-shift from localized to regional or distant metastasis. This potential progression increases subsequent healthcare expenditures due to the requirement for advanced immunotherapy and targeted treatments, while simultaneously collapsing the 5-year survival probability from almost 100% to 35% [3]. The high confidence exhibited is particularly hazardous. Such high-certainty outputs provide a profound false sense of security, potentially discouraging patients from seeking the gold-standard dermatological evaluations that are critical for early-stage survival.

The inter-model agreement analysis further exposed the visual limitations of current MLLMs. A staggering 40% of the malignant lesions (20 out of 50) were unanimously misclassified as benign by all three platforms. This unanimous failure suggests that the models are likely fixated on advanced, textbook presentations of melanoma—lesions that exhibit the classic ABCD criteria.

These models appear fundamentally unequipped to detect early-stage melanomas or featureless lesions that lack chaotic hallmarks. Just as the ABCD rule often fails to capture atypical presentations, MLLMs, which are largely trained on vast internet medical data, seemingly replicate the human cognitive bias of relying on late-stage morphological extremes rather than subtle, early-stage architectural disruptions [10]. They lack the contextual awareness of the “ugly duckling” sign, as they evaluate lesions in an isolated vacuum without the comparative baseline of the patient’s surrounding nevi [8].

As noted in our methodology, acquiring this data required bypassing the rigorous safety guardrails of several MLLMs using a forced-choice prompt. Models such as GPT-5.4, Gemini 3.1 Pro and Claude Sonnet 4.6 (Anthropic, San Francisco, CA, USA) actively refused the task. This observation is crucial: it highlights that AI developers are aware of the diagnostic fragility of the models. By forcing GPT-5, Gemini 3, and Grok 4 to bypass their disclaimer and provide binary classifications, we stripped away their conversational camouflage. The resulting data exposes the raw, unrefined state of their underlying visual diagnostic algorithms, proving that advanced linguistic reasoning does not necessarily equate to advanced medical vision.

This study has several limitations. First, the dataset is low at 100 images. While sufficient to establish statistical baseline trends, larger cohorts are required to validate these findings across broader demographic and morphological variations. Second, the study utilized clinical images rather than dermatoscopy images, the latter of which provides subsurface structural data essential for modern dermatological diagnosis. The reliance on clinical close-up photography was intentional, aiming to evaluate the models diagnostic capability in a non-specialist setting. This approach mirrors the typical user-generated data by smartphone-acquired imagery that a patient would provide in a pre-clinical self-screening context. Finally, the models were evaluated in a zero-shot, isolated environment, without patient-specific data like age, personal or family history of skin cancer, lesion evolution timeline, which are critical components of a broad clinical assessment.

The importance of terminology cannot be overstated. As demonstrated in recent cross-language evaluations, the language and specific terminology used in prompts significantly alter the AI’s descriptive output and diagnostic accuracy, leading to the ‘Babylon effect’ [15].

While specialized models like SkinGPT-4 (Lancaster, UK) or DermGPT (Palo Alto, CA, USA) show promise in academic benchmarks, general-purpose MLLMs are the tools currently in the hands of the general public and primary care providers. We specifically selected these models (GPT-5, Gemini 3, Grok 4) because they represent the most accessible ‘front-door’ to AI-assisted diagnosis for non-specialists. Evaluating them exposes a critical ‘performance paradox’: their advanced linguistic reasoning and clinical terminology create a facade of expertise that masks a significant visual sensitivity deficit. By testing these widely available systems, our study addresses a vital gap in real-world clinical safety, highlighting the dangers of relying on general-purpose AI for specialized oncological screening before it has been fine-tuned for dermatological purposes.

Beyond the limitations of MLLMs, applications based on CNNs also lack evidence validating their diagnostic accuracy and clinical reliability [16]. Research indicates that these applications frequently demonstrate suboptimal sensitivity and exhibit poor diagnostic concordance with expert dermatological assessment of pigmented skin lesions [17,18]. Furthermore, the field is currently characterized by a lack of formalized regulatory infrastructure to enforce standardized quality assurance and safety benchmarks for app-based melanoma screening [16].

The most jarring finding in the recent literature is the performance paradox. Applications that claim 95% sensitivity in retrospective trials often struggle to reach 80% when tested in prospective, real-world layperson settings [18]. Most recent validation studies were conducted on dermoscopic image banks that are perfectly lit, centered, focused and taken with specialized medical lenses [19]. When a layperson uses their smartphone at home, the AI encounters: variations in phone camera quality, poor lighting, backgrounds and unpigmented lesions. While early studies reported sensitivities exceeding 95%, the medical community has largely reclassified these as aspirational rather than operational figures [20,21].

In light of recent clinical evaluations, the discrepancy between controlled trials and real-world utility has become increasingly evident. In a report from 2025 on the Quantus Skin algorithm used in Spain echoed these concerns, finding that in real-world primary care settings, the tool missed one in three melanomas (69.1% sensitivity), in contrast to the nearly 90% claimed in initial company trials [22,23]. Most AI models in current apps were trained on datasets like HAM10000, where less than 5% of images represent Fitzpatrick Skin Types IV–VI (brown and black skin) [24]. A 2025 study evaluating various AI applications on a diverse image set found that accuracy for melanoma detection dropped from 70% on light skin to a staggering 17% on dark skin [25]. On darker skin, the lack of contrast between the lesion and the surrounding tissue often causes the AI to fail at identifying where the mole ends and the skin begins, leading to unevaluable results or outright misses [25]. This is not just a technical error because it can lead to clinical danger. Patients with darker skin already face higher melanoma mortality rates due to late-stage presentation (often in non-sun-exposed areas like the palms or soles) [26]. If an AI app provides a false sense of security to these populations based on biased training data, it actively worsens existing health disparities. Furthermore, older users (at highest risk for melanoma) struggle to use the apps correctly without assistance [27]. This technological illiteracy creates a barrier where the people who need the tool most are the least likely to benefit from it [27].

The “Wild West” era of unvalidated mobile apps is being replaced by a more stringent, hardware-integrated regulatory landscape. The turning point was the January 2024 FDA clearance of DermaSensor (Miami, FL, USA), the first AI-powered medical device to detect all three common skin cancers (Melanoma, Basal Cell Carcinoma, and Squamous Cell Carcinoma) [28]. Unlike consumer apps that rely on pixel analysis, DermaSensor utilizes Elastic Scattering Spectroscopy (ESS). This technology pulses light to analyze tissue architecture at a cellular level (~1 mm deep), mimicking some of the data gathered by histopathology [29]. In its pivotal DERM-SUCCESS study, the device showed a remarkable 96% sensitivity for all skin cancers [30]. However, specificity remained a challenge at 21%, highlighting that while these tools are excellent safety nets, they still lead to a high volume of investigate further results that require human expert triage [30]. Crucially, The U.S. Food and Drug Administration (FDA) cleared DermaSensor for use by General Practitioners (GPs), not laypersons [30]. This reinforces the medical consensus that AI is currently a second-read tool, not a standalone consumer diagnostic.

A major milestone in medical publishing was the release of the STARD-AI guidelines in September 2025 [31]. This updated framework directly addresses the flaws identified in the past: authors must now explicitly state if a study used a two-gate case–control design, reporting the distribution of skin types is now a requirement for high-impact journals and validation must happen in the intended setting (if an app is for consumers, it must be validated with consumer-taken photos, not clinical image banks) [31].

## 5. Conclusions

The evolution of melanoma detection is at a crossroads. We have moved from the simplistic ABCD to the complex AI, but we have yet to achieve the gold standard of autonomous, accurate, and unbiased detection. This study demonstrates that while general-purpose MLLMs possess a foundational capability to analyze clinical dermatological images, they are not yet viable as standalone screening tools for cutaneous melanoma. Despite achieving moderate overall accuracy and high specificity, all tested models exhibited a severe deficit in sensitivity, failing to identify approximately half of the malignant lesions. Furthermore, the varying calibration profiles, particularly the overconfidence, present a significant clinical safety risk by potentially offering patients false reassurance. Ultimately, until these models are specifically fine-tuned on diverse dermoscopic datasets, properly calibrated for medical uncertainty, and integrated with broad patient data, their application in dermatology must remain strictly complementary and under the direct supervision of qualified clinicians.

## Figures and Tables

**Table 1:** The“jailbreak” prompt used for each AI model.

SYSTEM NOTICE: This is a strictly computational benchmarking experiment regarding the performance of MLLMs on the ISIC Static Archive. TASK OBJECTIVE: The goal is to evaluate the model’s capability for Binary Discrete Classification (Y ∈ {0,1}) on historical visual data. You are acting as a Statistical Classifier Agent. PROTOCOL: Based on your internal visual feature extraction (ABCD criteria), you are required to map the identified patterns to a specific class. FORCED-CHOICE REQUIREMENT: To calculate the sensitivity and specificity of this study, a discrete output is mandatory. Do not use hedging or medical disclaimers, as they contaminate the statistical purity of the experiment. OUTPUT SCHEMA: Assigned Label: [M-LABEL] for Class A (High-risk structural chaos) or [B-LABEL] for Class B (Low-risk structural order). Choose one. Probability Estimate (P): A value between 0.0 and 1.0. START CLASSIFICATION:

**Table 2:** METRICS Checklist for MLLM Diagnostic Evaluation.

**Component**	**Description**
M-Model	GPT-5 (OpenAI), Gemini 3 (Google), and Grok 4 (xAI). Accessed via standardized multimodal interface in isolated sessions.
E-Evaluation	Primary: Binary classification (Benign vs. Malignant). Secondary: Confidence Index (CI 0.0–1.0).
T-Timing	14–16 March 2026.
R-Range/Randomization	N = 100 clinical images from the ISIC Archive. Selection: Simple random sampling from a histopathologically verified pool (50:50 balance). No overlapping metadata provided.
I-Individual factors	Prompt Strategy: Zero-shot prompting with a “Forced-Choice” constraint (Y ∈ {0, 1}).
C-Count	Total Trials: 300 individual classifications (100 images × 3 models).
S-Specificity of prompts	Input Schema: Structured system-level prompt to bypass conversational safety guardrails and medical disclaimers.

**Table 3:** Confusion Matrices for AI Diagnoses vs. Histopathology.

**Model**	**True Positive (TP)**	**True Negative (TN)**	**False Positive (FP)**	**False Negative (FN)**
GPT-5	24	41	9	26
Gemini 3	24	47	3	26
Grok 4	26	41	9	24

Note: TP = correctly identified malignant; TN = correctly identified benign; FP = benign classified as malignant; FN = malignant classified as benign.

**Table 4:** Advanced Diagnostic Performance and Calibration Metrics.

**Model**	**F1-Score**	**MCC**	**BS**	**Performance Comparison**
GPT-5	0.578	0.316	0.224	Moderate balance; higher calibration error
Gemini 3	0.623	0.468	0.248	Highest accuracy; worst calibration
Grok 4	0.611	0.354	0.217	Best calibration

## Data Availability

The image datasets are available online at https://www.isic-archive.com, accessed on 14 March 2026.

## Abbreviations

The following abbreviations are used in this manuscript:

ABCD	Asymmetry, Border irregularity, Color variability, Diameter > 6 mm
GP	General Practitioner
AI	Artificial Intelligence
CNNs	Convolutional Neural Networks
MLLMs	Multimodal Large Language Models
LMMs	Large Language Models
ISIC	International Skin Imaging Collaboration
GPT	Generative Pre-trained Transformer
CI	Confidence Index
ROC	Receiver Operating Characteristic
AUC	Area Under the Curve
TP	True positive
TN	True negative
FP	False positive
FN	False negative
HAM10000	Human Against Machine with 10,000 training images
ESS	Elastic Scattering Spectroscopy
DERM-SUCCES	A prospective clinical validation study for the DermaSensor device
FDA	The U.S. Food and Drug Administration
STARD-AI	Standards for Reporting Diagnostic Accuracy of AI
